# Calcium Nutrition of Broilers: Current Perspectives and Challenges

**DOI:** 10.3390/ani13101590

**Published:** 2023-05-09

**Authors:** Laura Shiromi David, M. Naveed Anwar, M. Reza Abdollahi, Michael R. Bedford, Velmurugu Ravindran

**Affiliations:** 1 Monogastric Research Centre, School of Agriculture and Environment, Massey University, Palmerston North 4442, New Zealand; L.David@massey.ac.nz (L.S.D.); naveed.anwar@vdbpoultry.co.nz (M.N.A.);; 2 Van Den Brink Poultry Ltd., Christchurch 7677, New Zealand; 3 AB Vista, Marlborough SN8 4AN, UK; mike.bedford@abvista.com

**Keywords:** calcium, ileal digestibility, limestone, phosphorous, requirement

## Abstract

**Simple Summary:**

Calcium (Ca) is essential for the skeletal growth and a plethora of other functions in broilers. Calcium is closely related to phosphorus (P) in terms of absorption and postabsorptive utilisation of both minerals. However, the oversupply of Ca and its potential antinutritive effects in commercial broiler diets are currently receiving attention. In recent decades, considerable attention has been directed towards the P nutrition of poultry because P is the third most expensive nutrient in poultry diets and is a major contributor to environmental pollution. To improve P utilisation and conservation, the poultry industry is moving to a digestible P system from the historically used nonphytate or available P. Thus, there is an urgent need to develop a digestible Ca system in order to meet the requirement of birds and generate accurate digestible Ca to digestible P ratios. During the last decade, considerable progress has been made to define the availability of Ca and P in terms of ileal digestibility. The specific aim of the present overview was to highlight the recent advances in the measurement of ileal Ca digestibility of Ca sources and digestible Ca requirement of broilers. Aspects of homeostatic control mechanisms, Ca sources and the factors influencing Ca digestibility are also addressed.

**Abstract:**

Calcium (Ca) plays an essential role in poultry nutrition as 99% of Ca is located in birds’ skeletal system. However, oversupply of Ca rather than deficiency of Ca is the current concern in commercial broiler diets. Calcium is an inexpensive dietary nutrient due to the cheap and abundant availability of limestone, the major Ca source; therefore, little attention was given to the oversupply of Ca in the past. The recent shift in the use of digestible P in broiler feed formulations has necessitated a closer look at digestible Ca, as Ca and P are interrelated in their absorption and postabsorptive utilisation. In this context, data on ileal digestibility of Ca and P in ingredients has been determined. Preliminary data on the digestible Ca and digestible P requirements for the different growth stages of broilers have also recently become available. The present review focusses on these recent advances in Ca nutrition. In addition, aspects of homeostatic control mechanisms, different Ca sources and factors influencing Ca digestibility in poultry are covered.

## 1. Introduction

Humphry Davy, a British chemist, was the first to isolate and investigate the chemistry of calcium (Ca) and to recognise it as an essential component of bone in 1808. Ever since, it has been the subject of a voluminous amount of research in both human and animal nutrition. Today, the biological significance and economic importance of Ca in animals are intuitive. Calcium is the most abundant mineral in the body and is essential for the skeletal health and animal welfare. Over 99% of Ca in the animal body is located in the skeleton [1] where it exists in the form of hydroxyl–apatite in a ratio of 2:1 with phosphorus (P). Calcium is also important for a wide range of functions in the body, for example, blood clotting, muscle contraction, nerve impulse transmission, enzyme activation, metabolic reactions, protein synthesis and maintenance of osmotic and acid-base balance [1].

The absorption and utilisation of Ca and P are mutually dependent, and these two minerals should be considered together in animal feeding. For maximum efficiency of utilisation, the supply of Ca and P must be matched as closely as possible to the requirements of the animal. Studies have demonstrated that increasing concentration of one mineral (Ca or P) decreases the absorption of the other [2,3].

In recent decades, considerable attention has been directed towards the P nutrition of poultry for the following reasons: (i) P is the third most expensive component in poultry diets, (ii) its supply is nonrenewable and (iii) P excretion is a major contributor, after nitrogen, to environmental pollution [4]. On the other hand, Ca nutrition and metabolism have been relatively neglected, because of the abundance of Ca in the earth’s crust. The supply of Ca is inexpensive and its excretion into the environment does not represent a threat to the environment. To improve the utilisation of P and conserve it for the future, the poultry industry is currently moving to a digestible P system from the historically used available (nonphytate) P system. Thus, there is a need to develop a digestible Ca system in order to derive more precise requirements and ultimately provide accurate digestible Ca to digestible P ratios for each growth or production phase of the bird. Some progress has been made during the last decade in defining P and Ca availability in terms of ileal digestibility [4,5,6]. As new research emerges, guidelines need to be updated or revised to maintain relevance. It is not the intention of this paper to provide a review of early studies on Ca nutrition of broilers, but rather to emphasise more recent research on the measurement of ileal Ca digestibility and topical work on establishing the digestible Ca requirements. The overview will also address aspects of homeostatic control mechanisms, the various Ca sources and factors influencing Ca digestibility in poultry.

As noted above, it is difficult to accomplish an analysis of Ca nutrition as a subject distinct from P nutrition. The question of Ca and P metabolism is a circle with no convenient point of interruption. In the present review, the focus will be of Ca and its complicated interactions with P will not be covered unless deemed relevant. Phosphorus nutrition is a subject unto itself and, a separate discussion on Ca and P interactions is beyond the scope of the current work. Some relevant reviews include van der Klis and Versteegh [7], Veum [8], Adedokun and Adeola [9], and Proszkowiec-Weglarz and Angel [10].

The Ca metabolism and bone biology of laying hens conspicuously differ from those of broilers because of their additional role in egg formation and shell quality. The aspects of Ca nutrition in layers are exclusive and complex, and will not be covered herein. The readers are directed to publications by Etches [11], Whitehead [12], de Vries et al. [13], Nys and Guyot [14], Kim et al. [15] and Korver [16].

## 2. Calcium Homeostasis

Homeostasis is a simple concept long appreciated by physiologists and is defined as a state of equilibrium in the body with respect to various functions and to the chemical composition of fluids and tissues; this enables animals to perform normally with variable nutrient intakes [17]. A fundamental understanding of Ca homeostasis and how body Ca levels are controlled is crucial to decide what constitutes adequate dietary Ca levels for animals. Calcium is present in three forms in the body with over 99% of Ca being present in bones in the form of hydroxyapatite and the remaining 1% in intracellular and extracellular spaces. Extracellular Ca is only 0.1% of the total body Ca and is present in three forms, namely ionised Ca, Ca bound to proteins and Ca bound to anions. Ionised Ca is the only physiologically active form of Ca in birds [18].

There are four metabolic routes by which broilers adapt to variable dietary intakes of Ca.

(1) Changes in absorption

Change in absorption is a key route of adaptation for Ca to variable dietary Ca levels. For many years, based on isotopic studies, it was thought that Ca was absorbed in the duodenum and jejunum [19,20,21]. However, recent findings demonstrate that the jejunum and ileum are the major segments of Ca absorption in poultry and, in fact, Ca digestibility was negative in the duodenum [2].

At this point, the conflict in the literature over the terms ‘absorption’ and ‘digestion’ must be noted. For minerals, including Ca, absorption is perhaps the most appropriate scientific term because there is no enzymatic hydrolysis. Instead, solubilisation is the first step prior to transport across the intestinal epithelium. In poultry, the solubility of Ca in the gastrointestinal tract is pH-dependent and the rate of dissolution is influenced by particle size. The proventriculus and gizzard play important roles in this process. Solubility is negatively correlated to pH, with Ca being solubilised more at lower pH [22]. However the apparent disadvantage in the dissolution rate of large particulate Ca sources is somewhat offset by the fact that such large particles are retained longer in the gizzard. Once dissolved in the proventriculus and gizzard, the soluble Ca transits to the small intestine where the increment in pH threatens precipitation with anions such as P and phytic acid before it can be absorbed [23]. In this paper, consistent with current literature on feed evaluation, absorption is described by the terms ‘digestion and/or digestibility’.

(2) Excretion via urine and faeces

Excretion is another principal route for the homeostatic control of Ca. Since urine and faeces are voided together in poultry, collection of this composite allows the measurement of total Ca excretion and thus easy measurement of total retention of Ca. Faecal excretion represents undigested dietary Ca. Available data suggests that, within the dietary Ca ranges usually encountered in practical feeding of broilers, this component is significant, with reported values as high as 80% of intake [24] but generally in the range of 40–60% [25,26,27,28,29]. Urinary Ca excretion may become important only under specific conditions, mainly depending on dietary Ca and P levels. Separation and delineation of the undigested element excreted via urine, however, is difficult in poultry.

(3) Deposition in the skeletal system

In broilers, absorbed Ca is almost exclusively stored in the bone. As noted above, over 99% of body Ca reserves are in the bone, and the remaining 1% exist as free ions in soft tissues and fluids of the body [1]. When Ca is absorbed via the transepithelial membrane, it is quickly mixed with body fluids. If plasma Ca levels are within the normal range, then about half the ionised Ca is deposited in the bone, replacing the Ca released earlier, resulting in a constant exchange between blood and bone tissues [30]. The remaining 50% of plasma Ca that circulates is filtered into the tubules in kidneys and 70% of that can be reabsorbed from different parts of the kidneys [31].

Bone biology is a highly complex process [32]. During bone calcification, a framework of collagen is first laid down. Then calcium crystals from the blood fill the collagen framework, making the bones strong and flexible. Osteoblasts trigger and promote the crystallisation by the biological control to the secretion of various matrix proteins or enzymes [32].

(4) Endogenous Ca losses

Of all the routes of homeostatic control, endogenous Ca losses are the least understood. This is due mostly to difficulties in reliably defining and measuring this loss [33]. Endogenous Ca is secreted from the bile, pancreatic juice, gastric juice and damaged intestinal cell lining, and all of this is indicative of Ca ‘originating within the body’ [34]. To determine true Ca digestibility, the amount of endogenous Ca loss must be quantified. Anwar [5] determined the ileal endogenous Ca loss using Ca- and P-free diets in several assays and reported an average of 108 mg/kg dry matter intake (range, 84–127). These quantities, however, are negligible compared to the amount of undigested Ca in the ileum, and its application to true ileal Ca digestibility corrections makes little difference [5]. A similar scenario exists for P [4]. In contrast to these two minerals, the endogenous output of major nutrients such as lipids [35] and amino acids [36] are significant relative to undigested fractions, resulting in economically important differences between apparent and true digestibility values. Interestingly, endogenous losses ostensibly have considerable significance for several trace minerals [37]. For example, in sodium, manganese and zinc, endogenous losses may equal or even exceed true absorption, resulting in net negative retention over short periods of time.

Calcium homeostasis is regulated by hormones to maintain a narrow range of concentration for optimum function. Biochemical details involved in the homeostasis are recognised [8,9]. Intestinal Ca absorption, in ionic form (Ca++), is an essential process for the maintenance of Ca homeostasis. Two mechanisms, namely active (saturable) and passive (unsaturable), are involved in intestinal Ca absorption. Passive absorption is characterised by Ca ion movement from the intestinal lumen to blood circulation along the chemical gradient through spaces between cells [30]. Active absorption involves three steps, namely entry across the cell wall, diffusion through the cytoplasm and exit at the basolateral cell membrane. When Ca intake is either high or adequate, passive transport predominates. Bronner and Pansu [38] reported that active transport depends on vitamin D3 and takes place at low dietary Ca concentrations. They stated that active absorption takes place in the upper duodenum, but current evidence suggests that jejunum and ileum are the major sites of Ca absorption [2].

Maintenance of homeostasis usually involves negative feedback loops, which act to oppose the stimulus or cue that triggers them [17]. Calcium homeostasis in poultry is closely regulated by parathyroid hormone (PTH), vitamin D3, calcitonin and related receptors in the small intestine, bone, liver and kidneys. Vitamin D3 is bound with protein and stored in fat or transported to the liver. This inactive form of vitamin D3 undergoes a double hydroxylation reaction by means of 25-hydroxylase and 1α-hydroxylase in the liver and kidney, respectively, resulting in the formation of 25-hydroxy D3 (25(OH)D3) and 1,25(OH)2D3) or active form of vitamin D3, respectively [39]. The enzymes responsible for this double-hydroxylation reaction are activated by PTH. Parathyroid hormone is released when the blood Ca concentration is low. Consequently, the active form of vitamin D3 increases in concentration and increases intestinal Ca absorption by stimulating a Ca transporter named calbindin. In addition, PTH increases Ca reabsorption from the renal tubes [10]. Calbindin is a Ca binding protein which transports Ca from the brush border membrane to the basolateral membrane of duodenal enterocytes. Calbindin D9k and Calbindin D28k are responsible for the transcellular diffusion of Ca in intestinal and renal tissues, respectively [10].

### 2.1. Excess Ca-An Obscure Anti-Nutrient

Oversupply rather than deficiency of Ca is often the problem in commercial broiler diets. Calcium is an inexpensive nutrient due to the cheap availability of limestone, the major Ca source, and, as a result, little attention is given to the oversupply of Ca. In recent Ca digestibility assays at Massey University (Table 1), the differences between calculated and analysed total Ca values of the experimental diets ranged from −2.1 to 1.8 g/kg, on an as-fed basis. Most of the analysed values were higher than the calculated values. Based on the analysis of 795 broiler and pig diets, Walk [40] also reported that the analysed Ca concentration is 25% above the expected. These differences represent an unresolved practical issue in commercial situations and in the measurement of Ca digestibility. Several factors contribute to this conundrum. Given the granular nature of Ca supplements, the predominant one is likely to be the errors in mixing and sampling, Differences in analytical procedures also contribute some variability. Another little-known fact is that Ca carbonate is the preferred carrier in most premixes (vitamins, trace minerals, drugs and nutritional additives). It is also added as a flow enhancer at the rate of 5 g/kg during the processing of soybean meal, particularly in the US mills [41]. Such inclusions are often not taken into account when formulating rations.

Excess Ca readily forms an insoluble complex with phytate, especially in the small intestine where pH is elevated [23] and can even precipitate with phosphorus. Additionally, high dietary Ca concentrations (when supplied in the form of limestone) increase the pH of gizzard digesta, resulting in reduced nutrient absorption [49] and growth performance [28,50,51] in broilers. The negative effects of high dietary Ca on the utilisation of P, along with those on several major nutrients and energy, make excesses of this mineral an antinutrient.

#### 2.1.1. Effects on P Utilisation

Studies have shown that increasing the Ca content of broiler diets reduces phytate-P hydrolysis and P digestibility [52,53,54]. The negative influence of high Ca concentrations on P digestibility was reported by Mutucumarana et al. [2] at different intestinal segments (duodenum, jejunum and ileum) of broilers. The same study also reported an aboral shift in the site of P digestion at higher Ca concentration where digestibility was higher in the upper ileum than in the jejunum. Similarly, Abdollahi et al. [51] reported a reduced ileal P digestibility in 21-day-old broilers fed high Ca (10.3–13.3 g/kg) diets. Reductions in ileal digestibility of P with increasing dietary Ca concentrations during different growth stages of broilers (days 1–10, 11–24 and 25–35) were reported by David et al. [28,29,55]. The reason for the reduced P digestibility is likely the formation of Ca-P/Ca-phytate complexes in the digestive tract due to excess Ca [23].

The influence of excess Ca on the utilisation of Ca itself has also been reported [29,56,57,58,59]. Atteh and Leeson [56] reported a reduced Ca retention and bone Ca in broilers fed 16 g/kg dietary Ca when compared to those fed 8 and 12 g/kg Ca. Similarly, Tancharoenrat and Ravindran [25] reported reduced total tract Ca retention coefficients (0.50 vs. 0.41 vs. 0.37) with increasing dietary Ca (7 vs. 10 vs. 13 g/kg, respectively). David et al. [29] also reported reduced ileal Ca digestibility in broiler growers with increasing Ca concentrations. However, in this study, excess Ca has negative consequences on Ca utilisation only when the P is deficient.

#### 2.1.2. Effects on Other Minerals and Major Nutrients

The influence of excess Ca on the utilisation of other minerals, nutrients and energy in poultry has long been known [60]; but the interest has been revived in recent decades [23], confirming the antinutritive effects of excess Ca (Table 2). High dietary Ca concentrations have been shown to reduce the availability of several biologically important minerals, including iron, magnesium, manganese and zinc, and protein [2,49]. This may be because excess Ca, most often achieved through increased limestone inclusion, increases intestinal pH which facilitates the precipitation of a number or essential cationic minerals with counter anions [49]. High dietary Ca also adversely affects the utilisation of fat [2,60,61] and metabolisable energy possibly though the formation of indigestible Ca soaps [56], with the eventual outcome of reduced growth performance and feed efficiency [25,62,63]. In addition, high dietary Ca increases leg abnormalities [64], possibly through impaired vitamin D absorption, which is reliant upon fat absorption. Tancharoenrat and Ravindran [25] reported a reduction in ileal fat digestibility from 63 to 55% when dietary Ca was increased from 7 to 13 g/kg. In the same study, the apparent metabolisable energy tended to be reduced from 3152 to 3105 kcal per kg of dry matter with the increasing dietary Ca. Shafey and McDonald [63] reported a negative effect of increasing dietary Ca (24 vs. 12 g/kg) on the retention of nitrogen. Avoiding excess Ca is particularly critical when other minerals are marginal in the diet.

#### 2.1.3. The Conundrum of Buffering Action of Limestone

Another complexity with limestone is its high buffering capacity (BC) or acid-binding capacity (ABC; [67]). This is a well-known fact to poultry nutritionists but generally ignored because of the difficulty in separating this effect from the other effects of excess Ca. Acid-binding capacity or BC is defined as the resistance of a feedstuff to pH reduction by gastric acid. Poultry feeds are usually in the alkaline range. Limestone is a strong buffer which can increase the digesta pH in the proventriculus and gizzard [68]. A high gastric pH adversely influences nutrient digestion by two mechanisms: (i) pepsin is not activated by pepsinogen at high pH; if the gastric pH is too high, protein breakdown will be impaired and (ii) alkaline pH increases the complexing of Ca and phytate-P, lowering the solubility and thus availability of both. Therefore, when Ca is supplied in excess in the form of limestone, the negative effects may be exacerbated. A highly buffered diet can also compromise the gastric and intestinal capacity to maintain an acidity level suitable to prevent the growth of harmful bacteria and maintain beneficial intestinal microbiome.

The ABC is measured as the amount of hydrochloric acid in milliequivalents required to lower the pH of 1 kg of feedstuff to pH 3.0, whereas BC is the amount of acid required to produce a unit change in the pH of a feed ingredient [67]. Table 3 provides a summary of data of determined ABC and BC of common ingredients used in broiler diets.

Finally, a brief mention is relevant for the Ca tolerance of laying hens. In contrast to nonlaying birds, laying hens can tolerate much higher dietary contents of Ca (10 vs. 30–40 g/kg) owing to specific adaptations for egg laying [12,15]. Laying hens have a unique calcium and bone metabolism to support egg production and eggshell formation. Modern strains of laying hens produce more than 320 eggs per year. Eggshell, in an average egg of 60 g, weighs 6 g and contains 2.4 g Ca; thus, hens require a considerable amount of Ca for the eggshell, driven by the metabolic need for higher Ca consumption.

### 2.2. Calcium-Specific Appetite in Broilers

Poultry has been shown to possess a specific appetite for Ca [69,70]. Wilkinson et al. [71] and Abdollahi et al. [51,72] confirmed the previously reported Ca-specific appetite in contemporary broilers. The Ca-specific appetite refers to the ability of birds to selectively ingest a Ca-rich source to meet their minimum Ca requirement [73]. This physiological self-regulatory mechanism for Ca intake may provide the opportunity to avoid the inclusion of Ca supplements in mixed diets and instead offer them separately. Separate Ca feeding may be a practical option to overcome the issue of Ca oversupply.

## 3. Calcium Sources

Over 80% of Ca in broiler diets is supplied by inorganic Ca sources because the Ca content of feed ingredients of plant origin are very low [74]. Limestone, oyster shell, monocalcium phosphate (MCP), dicalcium phosphate (DCP) and monodicalcium phosphate (MDCP) are the commonly used Ca sources, but limestone is the predominant supplement. Animal-based feed ingredients (meat and bone meal [MBM], bone meal, poultry by-product meal) and some plant-based ingredients (canola meal, soybean meal [SBM]) can also contribute reasonable amounts of Ca to poultry diets. The Ca content of common feed ingredients are summarised in Table 4.

### 3.1. Limestone

Limestone is the common Ca source used in poultry feed formulations because of its high Ca content. The primary component of limestone is calcite, but it may also be contaminated with Ca oxide (CaO), aragonite and dolomite (CaMg(CO_3_)_2_). Calcite and aragonite differ in the crystal arrangements of Ca carbonate (CaCO3). Different types of limestone are formed through a variety of processes such as precipitation, secretion by marine organisms, shells of dead sea creatures and cementation of sand or mud by calcite. Limestone can be classified into three categories based on its depositional environment such as platform, basin and geosynclinals [75]. An array of names are used for limestone based upon how the rock is formed, its appearance or its composition, along with other factors. Different colours of limestone (tan, grey, etc.) are due to impurities such as sand, clay, iron oxides and organic materials.

The Ca content of limestone is usually assumed to be 380 g/kg in most feed formulation matrices, but analysed values have been reported to vary from 304 to as high as 420 g/kg [76,77,78,79]. In a survey of 641 samples from 40 countries, Gilani et al. [80] found a range of 333 to 400 g/kg Ca, with an average of 379 g/kg. Based on chemical formula (CaCO_3_) and molecular weights, the maximum possible Ca content is 400.4 g/kg. Values higher than this may indicate analytical errors or contamination with CaO which has a higher Ca content. On the other hand, lower values are indicative of contaminants.

Limestone supplies up to 70% of the Ca in typical broiler diets. The availability of Ca in limestone was historically assumed to be 100% and therefore the limestone or purified forms of Ca carbonate has been used as the standard in the measurement of Ca bioavailability of other Ca sources. The measurement of Ca digestibility, however, is now the preferred method to express Ca availability and recent findings clearly demonstrate that Ca in limestone is not 100% digestible. Ileal Ca digestibility in limestone for broilers was determined to vary widely from 27 to 77%, depending on the source of limestone [48,79,81], particle size [42,44,81], in vitro solubility [81], dietary P content [42] and bird age [45]. The digestible Ca determined for a particular limestone sample, therefore, will not be applicable to other samples with different intrinsic factors, challenging the use of an average value in feed formulations.

#### 3.1.1. Limestone as a Source of Ca

Different sources of limestone contain differing Ca and mineral composition [48,79,80,82]. For example, dolomitic limestone contains high concentrations of magnesium (12%; [83]) when compared to calcitic limestone. Dolomitic limestone is derived from deposits of Ca carbonate combined with magnesium carbonate, whereas calcitic limestone is derived from deposits of primarily Ca carbonate. Magnesium competes with Ca for absorption sites and also excess magnesium binds with Ca in the intestinal tract, lowering availability of Ca for the animal. For these reasons, dolomitic limestone should be avoided in broiler diets.

Equally important, limestone samples of different origins also vary in their particle size and in vitro solubility. Furthermore, different limestones with similar particle size may have different in vitro solubilities [81,84]. Zhang and Coon [81] reported that the amount of limestone retained in the gizzard (5.90 vs. 3.81 g) was affected by limestone source which was related to the difference in in vitro solubility between the limestones. Anwar et al. [79] and David et al. [48] reported an influence of limestone sources on their ileal Ca digestibility. The study by Anwar et al. [79] found that the ileal Ca digestibility of three limestone samples with the same particle size in broilers were 0.54, 0.58 and 0.61. Such variation in Ca digestibility across limestone samples are inevitable due to a myriad of interacting factors affecting solubilisation and digestion.

#### 3.1.2. Limestone and Particle Size

Limestone particle size plays a major role in the Ca utilisation by poultry. Because coarser limestone particles are retained longer in the gizzard of poultry [44,81,85], they result in increased in vivo solubility and digestibility of Ca. Anwar et al. [42] reported a substantially higher true ileal Ca digestibility (0.71 vs. 0.43) for coarse limestone (1–2 mm) than fine limestone (<0.5 mm). Similarly, Kim et al. [86] reported a difference in the apparent ileal Ca digestibility between particulate (0.633 mm) and pulverised (0.063 mm) limestone samples of the same origin, with the digestibility in particulate samples being higher. These findings are in agreement with other studies [87,88,89].

#### 3.1.3. Limestone and Solubility

Solubility of limestone is determined as the percentage weight loss of limestone samples during an in vitro assay. Cheng and Coon [90] developed a technique to measure limestone solubility in vitro where the limestone samples (2 g) were allowed to solubilise for 10 min in 100 mL of 0.1 N hydrochloric acid (HCl), which was warmed for 15 min in a 42 °C water bath oscillating at 60 hertz. After 10 min, the contents were filtered gravimetrically through a Whatman 42 filter paper, dried at 70 °C for 10 h, cooled and weighed to determine the percent weight loss. Zhang and Coon [81] proposed the use of 200 mL of 0.2 N HCl instead of the 100 mL of 0.1 N HCl to avoid the excess buffering of the acid when a highly soluble limestone is tested. This method involves a very acidic solution (pH 0.76) and a one-time point (10 min) solubility determination, which is the widely used method for the measurement of in vitro Ca solubility. Recently, a dynamic model [86] with more than one solubility time point (5, 15 and 30 min) and of a solution that closely represents gizzard conditions (pH 3 and buffered) has been reported to correlate better with in vivo Ca digestibility when compared to the digestibility results obtained at an assay at one time point. These researchers developed prediction models for ileal Ca digestibility that included the solubility at 15 and 30 min time points as these time points were more relevant compared to that at a 5 min time point. Regardless, a robust correlation utilising multiple samples of limestone of varying particle sizes with in vivo Ca digestibility is yet to be established.

However, in vitro solubility of limestone has been reported to be inversely related with its in vivo solubility in laying hens [81,91]. The in vitro solubility of limestone samples greatly depends on the particle size and source of origin. For instance, coarse particles are known to less soluble in vitro when compared to fine particles [44,81] but as indicated above, such coarse particles are retained longer in the gizzard where the Ca is able to dissolve, which explains this inverse relationship on the other hand, different limestone sources of similar particle size were found to have different in vitro solubilities [84]. Gilani et al. [80] reported that the average in vitro solubility of 566 fine limestone samples at 5 min incubation (pH 3.0) ranged from 19 to 99%, whereas that of 75 coarse limestone samples at 30 min incubation (pH 3.0) ranged from 23 to 96%. The in vivo relevance of these assay results has not been ascertained thus far.

### 3.2. Other Inorganic Ca Sources

Oyster shell is another natural source of Ca carbonate. The Ca content of oyster shells is similar to that of limestone [74]. According to Reid and Weber [76], the Ca content of oyster shell varies from 344 to 390 g/kg. Augspurger and Baker [92] determined that the relative bioavailability of Ca from oyster shell is comparable to that of limestone and, as is the case for limestone, particle size and other factors influence Ca utilisation of oyster shells. Anwar et al. [44] reported ileal Ca digestibility coefficients of 0.33 and 0.56, respectively, for oyster shell with fine (<0.5 mm) and coarse (1.0–2.0 mm) particles. Scott et al. [93] reported that feeding Ca in the form of oyster shells is more effective than feeding the same amount of finely ground limestone because the larger particles that solubilised slowly resulting in higher Ca absorption. Overall, most studies report that feeding oyster shells has benefits on eggshell quality, similar to that of feeding limestone [94].

Dicalcium phosphate (CaHPO_4_) is Ca phosphate with its dihydrate, an odourless white-coloured powder. Dicalcium phosphate contains about 220 g/kg Ca and 190 g/kg P [74]. There are three forms, namely dihydrate (CaHPO_4_.2H_2_O, the mineral brushite), hemihydrate (CaHPO_4_.0.5H_2_O) and anhydrous (CaHPO_4_, the mineral monetite). Depending on the form of DCP, the P and Ca contents vary [95]. Dicalcium phosphate is produced by the neutralisation of Ca hydroxide with phosphoric acid, which precipitates the dihydrate as a solid or by reacting phosphoric acid with limestone. The purity of the DCP depends on the origin of the raw material and procedures employed in its industrial production. Dicalcium phosphate could also be produced through precipitation from bones and it is a coproduct from gelatine manufacture [96]. The relative biological value of bone-precipitated DCP was reported to be higher than commercial feed phosphates [96].

Monocalcium phosphate is an inorganic compound (Ca(H_2_PO_4_)_2_) and is commonly found as the monohydrate (Ca(H_2_PO_4_)2.H_2_O). Monocalcium phosphate is produced by treating Ca hydroxide with phosphoric acid and contains around 160 g/kg Ca and 220 g/kg P [74] MCP may contain some DCP, but more than 80% of the P should be derived from the MCP fraction to be classified as an MCP. Monodicalcium phosphate contains less than 80% P from MCP [95].

Tricalcium phosphate (TCP; Ca_3_(PO4)_2_) is a calcium salt of phosphoric acid, which is a white solid of low solubility. It exists as three crystalline polymorphs such as α, α’ and β. The α and α’ states are stable at high temperatures [97]. Tricalcium phosphate is produced commercially by treating hydroxyapatite with phosphoric acid and slaked lime. Tricalcium phosphate is also produced by heating a mixture of Ca pyrophosphate (Ca_2_P_2_O_7_) and Ca carbonate (CaCO_3_). Tricalcium phosphate occurs naturally in several forms such as rock, skeletons and teeth of vertebrate animals and milk. In some countries, TCP is used as the main inorganic phosphate supplement in poultry diets [98]. The production process of TCP from bones involves the degreasing of the bones in counter-flow with hot water (bone chips less than 14 mm). Then it undergoes continuous cooking with steam at 145 °C for 30 min at 4 bars and the separation of the protein broth from the hydroxyapatite (TCP) by centrifugation. The granulation of TCP is carried out after drying in a fluid bed with air at 200 °C. This TCP is not pure and, on average, composed of 750 g/kg hydroxyapatite, 170 g/kg gelatine, 4 g/kg fat and 4 g/kg moisture [99]. Feed grade TCP may be available in different trade names depending on the production process. For example, Hamdi et al. [100] used a TCP called lipocal, which is a TCP powder that has been treated with lecithin to reduce its interactions with other minerals and feed ingredients, especially in aqueous media. Rao et al. [101] used a TCP called Multifos, which is a deflourinated TCP derived from phosphate rock. Rao et al. [101] reported that the in vivo solubility of TCP (Multifos) was lower than that of DCP (Dynafos) or MCP (Biofos). Kwon and Kim [102] found that Ca digestibility was lower for TCP than for DCP and MCP.

Defluorinated phosphate (DFP; Ca_4_Na(PO_4_)_3_) is another mineral supplement used in poultry. Defluorinated feed phosphate is produced by removing the fluorine, which is toxic, from the phosphate rock. Natural phosphate rocks vary in fluorine content from less than 10 g/kg to above 40 g/kg [103]. Phosphate rock and sand are ground, mixed in a definite ratio and fed as a slurry to a rotary kiln. The material is treated at temperatures of 2700° to 2900° F. Water vapour is introduced at the hot end of the kiln and quick cooling of the product is undertaken. The discharged, compacted residue which is not fused, is ground and bagged. The product contains about 20% phosphorus pentoxide and 0.1% or less fluorine. The phosphate constituent is α-tricalcium phosphate [104]. Defluorinated feed phosphate is a good source of available P. Defluorinated feed phosphate is nonhydroscopic and noncaking powder or granules, light-brown to dark-brown in color and odorless. Compared to other mineral P sources, the DFP supplies Ca, P and sodium to animals at minimum concentrations of heavy metals and harmful components, and is highly soluble in the gut.

The use of lesser-known Ca sources has also been reported. Calcium citrate and Ca citrate-malate are reported to have similar relative bioavailability to that of limestone [92,105]. Although some other inorganic sources like agricultural grade phosphates and raw rock phosphates are cheaper than DCP, these contain high concentrations of heavy metals and can be toxic to animals [106].

### 3.3. Animal-Based Ca Sources

Animal protein sources, namely, MBM, fish mea, and poultry by-product meal also supply significant amounts of Ca in poultry diets. Meat and bone meal is a product of the rendering industry and an important organic Ca source, which contains an average of 103 g/kg Ca [74]. However, the Ca concentration of MBM varies widely depending on the source [43,107,108,109]. This variation in Ca concentration is due to the nature of the raw materials as well as the processing methods. Meat and bone meal is made from animal offal that is not suited for human consumption. Offal is cooked, defatted, sterilised and ground to obtain MBM. Depending on the proportions of bone and soft tissues used in the manufacture, the finished product is categorised as meat meal (containing > 55% crude protein and < 4.4% P) or meat and bone meal (containing < 55% crude protein and > 4.4% P). Similarly, fish meal is manufactured by cooking, pressing, drying and grinding the fish or fish waste that are not intended for human consumption. In the same way, poultry by-product meal is made by a similar rendering process.

### 3.4. Plant-Based Ca Sources

Plant-based feed ingredients are mostly used in poultry diets as an energy or protein supplements. They can, however, contribute only minimal amounts of Ca to the diet (Table 4). Among the plant-derived feed ingredients, canola meal (6.8 g/kg) and rice bran (7.0 g/kg) contribute are the richest sources of Ca for poultry diets [74]. The Ca content of SBM, the major protein source in poultry diets, is 2.8 g/kg [74] whereas those of cereals is negligible.

## 4. Ca Utilisation in Poultry

Historically, ‘total Ca’ was used for expressing Ca requirements and formulating poultry diets. Total Ca in a diet refers to the entire Ca content of the diet, regardless of the proportion available for absorption and utilisation by the animal. Calcium and P are interrelated in terms of their absorption and postabsorptive utilisation, and their requirements were expressed as total Ca and total P during 1950s. Although the expression of P requirement was changed to available P and nonphytate P in the NRC [110] and NRC [74], respectively, the expression of Ca requirement remained unchanged because of the lack of studies on Ca availability. The total Ca to available P and total Ca to nonphytate P ratios of broilers used during these periods ranged from 2.22 to 2.28 (up to 8 weeks of age) and 2.22 to 2.67 (depending on the growth stage), respectively [111]. Even though the terms available P and nonphytate P are different, they are normally used interchangeably. However, Mutucumarana et al. [112] reported that the P evaluation system based on nonphytate P is not precise as the values for true digestible P were higher than those for nonphytate P in maize and canola meal. Their observation suggested that a portion of phytate-bound P is utilised by broilers [53,113]. Regardless of the P term (available or nonphytate), the total Ca to P ratio is normally maintained at around 2:1 in broiler diets.

Clearly, the use of total Ca system is not precise [40,114], because there is the risk of Ca being oversupplied. It has been indicated that the total Ca concentrations of 6.5 and 6.0 g/kg were sufficient to meet the Ca requirement of broilers from day 1 to 14 and from day 15 to 49, respectively [114]. Applegate et al. [115] found that the recommended dietary total Ca concentration of 9 g/kg reduced the intestinal phytase activity and ileal phytate-P hydrolysis. Similarly, total Ca concentration of 10 g/kg has been shown to reduce the ileal digestibility of P in broilers [27].

According to Peeler [116], a confusing array of terms has been used in the literature to define the utilisation or availability of major minerals, such as percent utilisation, percent apparent digestibility, percent true digestibility, percent absorption, percent net retention, percent apparent availability, percent true availability and biological availability, but the meanings of these terms differ. Calcium availability was historically measured as bioavailability, using slope ratio assays. The slope ratio procedure involves feeding graded levels of Ca from a test ingredient below the requirement to induce an experimental deficiency response and the response criterion is plotted against Ca intake of animals fed diets containing the test ingredient diet and a known standard (limestone or calcium carbonate) that is presumed to be 100% available [117]. The response criteria are growth response (weight gain and feed conversion) and bone measurements (tibia ash, bone breaking strength and bone density).

In early research reports, the availability of Ca was determined to be high in Ca supplements. Dilworth et al. [118] reported that the Ca availability of Ca carbonate, low fluorine rock phosphate, defluorinated phosphate and soft phosphate were 100, 90, 92–95 and 68%, respectively. Calcium carbonate was found to have 100% biological availability, while the values for limestone, bone meal, TCP and DCP were 102, 109, 115 and 113%, respectively. However, dolomitic limestone and soft rock phosphate have relatively low Ca availabilities of 65 and 70%, respectively [119]. According to Reid and Weber [76], the Ca availability of ground limestone and oyster shell were 73–109 and 107.8%, respectively, in broilers. These available Ca concentrations, however, are rarely used in feed formulations, partly because total Ca content was deemed adequate. In addition, the phytate found in plant materials reduces Ca availability by binding Ca in phytate-mineral complexes [120,121].

Another term that has been used in the measurement of Ca utilisation is total tract Ca retention. Calcium retained over the total tract represents the proportion of dietary total Ca that is deposited in the body of an animal [122] and reflective of both digestive and postabsorptive utilisation of Ca [114]. Excess absorbed Ca which is not utilised in the animal’s body is excreted in the urine [123]. The measurement of retainable Ca requires the determination of Ca intake and output (faeces and urine). In poultry, faeces and urine (excreta) are excreted together which enable the Ca retention measurement easy.

The use of digestible nutrients in feed ingredients has now become the cornerstone of contemporary feed formulations that closely match the nutrient requirements for optimal and sustainable production. Ileal digestibility, the proportion of dietary Ca that is not recovered in the digesta at the terminal ileum, is currently accepted as the preferred method of digestibility measurement. The digestibility assays in poultry should be based on the analysis of ileal digesta rather than of excreta, because of the variable and modifying effects of hindgut microflora and possible urine contamination [124].

Calcium digestibility can be presented as diet digestibility and ingredient digestibility. In ingredient digestibility studies, the particular ingredient or Ca supplement serves as the sole source of Ca, whereas in diet digestibility the Ca assumes that Ca from individual ingredients in the diet is additive. In general, it is presumed that the amount of digestible nutrients in a diet is similar to the sum of digestible nutrients obtained from each dietary ingredient used in mixing that diet [125]. However, unlike the studies on additivity of different digestible nutrients [126,127,128,129,130], studies on the additivity of digestible Ca in poultry are scant. In the only available study, Zhang and Adeola [131] reported that the true ileal Ca digestibility in limestone and DCP are additive in semi-purified diets for broilers.

The available data on the ileal Ca digestibility of Ca sources are summarised in Table 5. Walk et al. [132] also highlighted some of these recent findings on Ca digestibility in broilers, which need be considered along with the current data.

Digestibility of nutrients can be expressed as apparent and true digestibility. True digestibility is measured by correcting the apparent digestibility for endogenous losses. Endogenous losses can be categorised into basal and specific endogenous losses based on dietary independence and dependence, respectively. Since basal endogenous losses are easier to measure than total (basal plus specific) endogenous losses, basal endogenous losses are used to calculate the true digestibility [135].

## 5. Factors Affecting Ca Digestibility

Calcium digestibility in poultry is influenced by dietary, bird and physiological factors as summarised in Table 6.

### 5.1. Particle Size, Ca Source and Solubility

Particle size is known to influence the Ca digestibility, but its effects are interlinked with Ca source and in vitro solubility. For instance, a Ca source with fine particles has higher in vitro solubility than the coarse particles of the same Ca source [81,84,87]. On the other hand, the differences in the Ca digestibility of different Ca sources are to be expected due to variable inherent characteristics. Table 7 summarises the ileal Ca digestibility of Ca sources with different particle sizes and in vitro solubilities.

### 5.2. Age and Ca Digestibility

Unlike major nutrients, reports on the effect of age on mineral digestibility in poultry are limited. Most of the available studies have focused on the first three weeks of life, with contradictory findings. Some have reported a decreasing trend in Ca digestibility with advancing age of broilers [133,148,149,150]. In contrast, Morgan et al. [155] reported that the ileal Ca digestibility of a maize-SBM diet was higher at week 2 compared to that at week 1. David et al. [47] observed that the apparent ileal Ca digestibility of limestone linearly decreased from day 7 (0.51) to day 42 (0.27) in broilers. Table 8 summarises the published data on the effect of broiler age on the Ca digestibility in broilers. These findings highlight the significant influence of age on the Ca digestibility and indicate that the use of digestible Ca values are not interchangeable among different age groups and age-dependent values may need to be considered in feed formulations.

### 5.3. Phytate, Phytase and Ca digestibility

Phytate, the storage form of P in plant seeds, is a salt of phytic acid (myo-inositol 1, 2, 3, 4, 5, 6-hexakis dihydrogen phosphate, IP6) and occurs as a complex of Ca or magnesium with myo-inositol [156]. The negative effects of phytate on nutrient utilisation in poultry was not appreciated until the commercial introduction of microbial phytases in the 1990s. Today, the effectiveness of microbial phytase on hydrolysing phytate-bound P and improving P bioavailability in poultry diets is well accepted and the inclusion has become common place.

Phytase not only improves the digestibility of P, but also of Ca and other minerals [23,142], because phytase hydrolyses the phytate-bound Ca to make it available for absorption in the broiler’s digestive tract. Inclusion of phytases in the diets of poultry has been shown to increase Ca digestibility [23,53,88,142,157]. Qian et al. [158,159] reported that the apparent Ca retention was increased linearly in turkeys, with increased phytase doses (0, 300, 600 and 900 FTU/kg). Similarly, Walters et al. [157] reported that increasing phytase doses (250–3000 FTU/kg) increased Ca digestibility in broilers. Furthermore, high concentrations of Ca inhibit the efficacy of both endogenous and exogenous phytases [115,160,161]. Presumably, this could be a consequence of insoluble Ca–phytate complex formation. In an in vitro assay, Tamim and Angel [52] reported that addition of 5 g/kg Ca from limestone reduced phytate–P hydrolysis at both pH 2.5 and pH 6.5. According to Akter et al. [162], dietary Ca of 10 g/kg diet negatively affects the phytase activity and phytate–P hydrolysis in broilers, especially when the nonphytate P is low.

Given that supplemental phytase is now added routinely in broiler diets, establishing the digestible Ca of diets with a background of phytase is crucial. Evidence suggests that the phytase effects on Ca digestibility may be dose-dependent. The effect of normal doses (500 FTU/kg) of phytase on Ca digestibility is contradictory. In some studies, phytase addition improved Ca digestibility [88,141,142], whereas little or no benefit was observed in others [58,163]. The doses higher than the recommendation are known as superdoses, which are currently being used by the industry and reported to improve the growth performance and nutrient utilisation over the normal doses [164]. Phytase has been known to hydrolyse not only the phytic acid (IP6) but also the lower phytate esters (IP5 to IP1), especially at superdoses. Administration of superdoses have been found to prevent the buildup of lower phytate esters in the digestive tract, thereby reducing the antinutritive effects of phytate further [164,165,166]. There is overwhelming evidence that support consistently greater responses in ileal Ca digestibility with superdosing [142,167,168,169,170,171].

David et al. [134] examined the ileal Ca digestibility in SBM and canola meal at three doses of phytase (0, 500 and 2000 FTU/kg). In this study, added phytase increased the true ileal Ca digestibility in both the SBM and canola meal, and the effect was more pronounced for the canola meal. This finding indicates that more phytate-bound Ca and P were released by phytase in the canola meal, which may be due to the relatively high phytate concentration of canola meal. The superdosing of phytase (2000 FTU/kg) increased the Ca digestibility in canola meal and SBM twofold compared to the normal phytase dose (500 FTU/kg).

Effects of supplemental phytase on Ca digestibility are also complicated by dietary Ca and P levels, as discussed in detail by de Lange and Kwakernaak [172]. An analysis by these researchers revealed that the influence of phytase on Ca digestibility is small in diets adequate in Ca. On the other hand, there is increased Ca absorption with added phytase in P-deficient diets. It was proposed that that the best design to study the relationship between Ca and phytase is by comparing graded levels of Ca in adequate P diets using tibia ash and ileal Ca digestion as response parameters.

### 5.4. Methodology

The methodology for Ca digestibility measurements is still in the developmental stage and currently there is no accepted standard procedure. In feed evaluation, three approaches namely, direct, difference (substitution) and regression methods are available for digestibility measurements [173]. The test ingredient serves as the sole source of nutrient in the assay diet in the direct method. In the difference method, a basal and a test diet are formulated, and the test diet comprises a mixture (usually 50:50) of the basal and test ingredient. The digestibility of the nutrient in the test ingredient is then determined based on the difference in digestibility between the two assay diets and the concentration of the specific nutrient in the test diet [174]. Regression method involves establishing a linear relationship between nutrient output in ileal digesta and dietary nutrient input by formulating diets with graded concentrations of the nutrient from the test ingredient. This method is costly and usually not preferred.

A majority of the published studies to date on the measurement of ileal Ca digestibility of Ca sources in broilers have used the direct method because of its simplicity. The method, however, requires further refinement. For example, David et al. [46] found that the Ca digestibility of limestone, MBM, MCP and DCP was influenced by the composition of the basal diet composition, with digestibility being higher in maize-based diet than in purified diet [46]. The contribution of Ca from maize (<0.02%) to maize-based diets is negligible and, therefore, the values determined with maize-based diets relate to the Ca source.

Another aspect to be considered is the length of dietary adaptation. A study by David et al. [46] found that the ileal Ca digestibility was affected by the dietary adaptation length with a higher digestibility coefficient at 24 h (0.65) of adaptation length, steady digestibility values between 72 (0.46) and 120 (0.44) hours and a decrease at 168 h (0.36). The findings indicated that the methodology for Ca digestibility measurement in poultry could use a 3–5 day adaptation length as the standard practice. Proszkowiec-Weglarz and Angel [10] also reported a reduced apparent ileal Ca digestibility in broilers with increased dietary adaptation length. Similarly, Anwar et al. [45] found that the apparent and true Ca digestibility of DCP were higher at 24 h of adaptation when compared to those at 48 and 72 h. The true Ca digestibility values reported for DCP at 24, 48 and 72 h of adaptation length were 0.45, 0.36 and 0.35, respectively [45]. In the same study, however, the adaptation length had no influence on the Ca digestibility of MCP.

Perhaps the most confronting aspect is the Ca:P ratios to be used in assay diets. Dietary Ca to P ratio is known to influence the absorption and utilisation of both minerals [9]. Maintaining an appropriate Ca to P ratio is important for the correct measurement of Ca digestibility. Gautier et al. [59], experimenting with a wide range of total Ca to nonphytate P ratios (from 1.3:1 to 5.3:1), reported reduction in Ca digestibility with widening ratios. Amerah et al. [139] found a higher ileal Ca digestibility at a 1.4 total Ca to available P ratio than at a 2.1 ratio (0.55 vs. 0.46). Anwar et al. [42] similarly reported that widening the Ca to nonphytate P ratios, from 1.5 to 2.5, in broiler diets reduced the true ileal Ca digestibility (0.65 and 0.49, respectively) of limestone. A ratio of 2:1 has been used in most Ca digestibility assays to date. While achieving this ratio is possible in Ca sources with no P (e.g., limestone) and with lower concentrations of P than Ca (e.g., DCP) by the inclusion of monosodium phosphate. On the other hand, a 2:1 ratio is not achievable in Ca sources (e.g., MCP) containing greater concentrations of P relative to Ca. The alternate option in such cases is the use of difference method.

A comparison of different methodologies was reported by Anwar et al. [45]. These researchers found that the Ca digestibility in DCP was lower with difference (0.21) and regression (0.13) methods than the direct method (0.34). It was concluded that the direct method is more appropriate for Ca digestibility assays.

## 6. Digestible Ca Requirement of Broilers

Despite the evidence that Ca in Ca sources, including limestone, is not 100% available, the Ca requirement of broilers is still being considered on total Ca basis. For example, the latest recommendation of Ca and P requirements for Ross broiler starters [175] are listed as 9.5 g/kg total Ca and 5.0 g/kg available P, respectively. Furthermore, recent interest in shifting towards the use of digestible P in poultry feed formulations has necessitated more attention on digestible Ca requirement.

Two sets of studies have investigated the digestible Ca requirements of broilers (Table 9 and Table 10). One was with Ross 308 broilers and the other with Arbor Acres. In the Ross study, weight gain and tibia ash of broiler starters, growers and finishers were maximised at 5.0, 3.5 and 3.5 g/kg SID P concentration, respectively. Corresponding SID Ca requirements for maximum weight gain were 3.32, 3.05 and 3.5 g/kg, respectively (corresponding to 7.0, 6.1 and 6.4 g/kg total Ca approximately), and for maximum tibia ash were 4.51, 3.69 and 3.0–3.5 g/kg, respectively (corresponding to 9.2, 7.3 and 5.5–6.4 g/kg total Ca approximately), at respective SID P concentrations. The equivalent total Ca requirement for maximum tibia ash in broiler starters, growers and finishers was 4, 16 and 18–29%, respectively, lower than the Ross 308 [176] recommendations. Overall, the findings suggested that the birds require Ca and P for bone–tissue synthesis beyond the needs for soft tissues.

In the Arbor Acres study, tibia ash of broiler starters, growers and finishers were maximised at 5.30, 5.15 and 3.70 g/kg SID Ca concentration, respectively (corresponding to 10.7, 9.9 and 7.8 g/kg total Ca approximately), at available P concentrations of 4.80, 4.40, and 3.90 g/kg, respectively (Table 10).

As could be noted, differences were observed in the Ca requirement estimates between the two studies. Possible reasons for the discrepancy may include the following. First, nonlinear prediction models (quadratic polynomial model, straight-broken line and quadratic-broken line regressions) in Arbor Acres studies were employed as against response surface model in the Ross studies and likely to be the primary contributor. Second, Arbor Acres assay diets contained phytase, protease and carbohydrase. These enzymes, especially the phytase, would have confounded the estimates. Third, the Arbor Acres studies considered available P values, but not the SID P. In addition, the SID requirement of both Ca and P should have been on a digestible basis. Fourth, the SID Ca requirement was estimated at a single P concentration (4.8, 4.4 and 3.9 g/kg available P for starters, growers and finishers, respectively) in the Arbor Acres studies.

Nevertheless, these data should be considered preliminary and should be useful in future investigations on digestible Ca requirements.

## 7. Conclusions and Future Perspectives

Calcium digestibility and utilisation in poultry are neglected areas of research. As already discussed, there are several key reasons for this neglect: (i) supply of Ca is inexpensive, (ii) its excretion into the environment does not pose any pollution issues like those by P, and (iii) until recently, the availability of Ca from Ca sources was assumed to be absolute. Perhaps the major complexity with Ca nutrition research is its close association with P. Several other factors (phytate, phytase, vitamin D) are also involved, and these are even more inextricably connected than previously thought.

The specific aim of the current review is to present updates on recent works on Ca and any detailed discussion on P was eluded for the sake of brevity. Progress in the measurement of Ca digestibility of Ca sources, various factors influencing Ca digestibility and digestible Ca requirements of broilers is provided. In addition, a critical discussion of limestone, the predominant Ca source, is also provided. These updates of Ca nutrition, however, raise more questions than answers. Among the unresolved issues, the following are worth mentioning and require immediate attention to make further progress in the Ca nutrition of broilers.

Limestone quality: When referring to research into Ca, we essentially imply limestone. In typical broiler diets, limestone supplies up to 70% of the Ca and hence its quality becomes critical. Current evidence has established that not all limestone is the same. Limestone samples differ widely in solubility and particle size, both of which have significant influence on Ca digestibility. In future studies, the correlation between in vitro solubility and in vivo digestibility needs to be confirmed. Then, rapid tests for the identification and separation on the basis of quality could be developed.

The buffering action of limestone may be another factor of concern in this equation. The ramifications of this property is yet to be clarified, but it undoubtedly plays some role in Ca digestibility. The effects of buffering action on digestion and broiler performance warrant future investigations.

Assay methodology: Variations observed in the ileal Ca digestibility in Ca sources among laboratories reflect, in part, the methodology used. Three basic methodologies, namely, direct, difference and regression methods, are potentially available for the measurement of nutrient digestibility. Of these, simplicity puts the direct method well ahead of the other two methods. As discussed earlier, the direct method is suitable for Ca sources containing no P (e.g., limestone) and those with narrower Ca:P ratios (e.g., DCP), as the Ca:P ratio could be adjusted to 2:1 by the addition of sodium monophosphate. For sources with high P content relative to Ca (wider Ca:P ratio), like the MCP, the direct method poses issues in balancing the Ca:P ratio to 2:1. The choice of methodology, therefore, may depend on the P content (or the Ca:P ratio) of the test source and studies are needed to explore this thesis. A collaborative study, similar to that conducted for amino acid digestibility [179], is proposed to test and develop a consensus protocol, which will minimise the variation and enable better comparison of data across laboratories.

Ca requirement studies: Determining an optimum Ca requirement is difficult for several reasons. Many interrelated factors influence the minimal Ca requirement and, P, Ca and vitamin D are among the most frequently examined factors. Phytate and microbial phytase are recent additions to this list. Of these, P is the first factor to be considered in a basic experimental design, especially since the Ca requirement may vary over a range of dietary P intakes. The two digestible Ca requirement studies aforementioned are useful starting points, but must be looked at as preliminary data. Hopefully further investigations will follow. Perhaps the concept of a narrow range of values, rather than a fixed value, may need to be considered a component of any recommendation. An added conundrum is that it is difficult to precisely answer the question about the digestible Ca requirement when the state of knowledge about digestible P requirement remains unsatisfactory.

Modelling of Ca and P metabolism: Biological systems are highly complex, due to the complicated nature of each of the components making up these systems. The construction of models allows a glimpse into the dynamic state of these components all at once. There is increasing interest among animal scientists in model building because of the usefulness of models in the examination of economic consequences of management decisions and in identifying research gaps. Building of a model for the growth and metabolic fates of Ca and P in broilers will be an essential first step in the understanding the intricate interactions between these two minerals.

Models [180,181] and a meta-analysis [182] of the metabolic fates of Ca and P have been reported for growing pigs, but none in broilers, largely because the field of Ca and P nutrition in poultry is data poor. The published data already available are insufficient to fully understand the intricate molecular mechanisms involved in their digestion, metabolism and partitioning to allow the construction of a model, as elegantly reviewed by Salisbury et al. [183].

## Figures and Tables

**Table 1:** Comparison of calculated and analysed dietary total calcium (Ca) concentrations (g/kg, as fed basis) in selected Massey University studies.

Reference	Calculated	Analysed	Difference ^1^
[42]	9.00	8.22–9.17	0.78 to −0.17
[43]	8.30	8.03–9.89	0.27 to −1.59
[44]	9.00	9.12–9.80	−0.12 to −0.80
[45]	9.01	9.48–11.12	−0.47 to −2.11
[46]	9.00	9.40–10.8	−0.40 to −1.80
[46]	6.08	5.80–6.90	0.28 to −0.82
[47]	9.00	8.90–9.90	0.10 to −0.90
[48]	8.00	8.40–9.50	−0.40 to −1.50
[28]	10.0	10.20–11.20	−0.20 to −1.20

^1^ Calculated dietary Ca concentration minus analysed dietary Ca concentration.

**Table 2:** Effects of excess calcium (g/kg) on the digestibility of nutrients in poultry.

Dietary Ca	Bird Class	Effect	Reference
12.0	Broilers	↓ P, N and fat digestibility	[2]
10.0	Broilers	↓ ileal P and protein digestibility	[27]
15.3, 21.8, 22.6	Broilers	↑ insoluble form of Ca, Mg, Zn and Fe	[49]
10.3, 13.3	Broilers	↓ ileal digestibility and retention of P	[51]
16.0	Broilers	↓ retention of fat, Ca and Mg	[56]
12.5	Broilers	↓ Ca Retention	[58]
16.0	Roosters	↓ digestibility of high-melting triglycerides and hydrogenated fats	[60]
20.0	Broilers	↓ iron retention	[65]
45.0	Layers	↓ body, egg and bone Ca retention	[66]

↓: Decrease; ↑: Increase; Abbreviations: Ca: calcium, Fe: iron, Mg: magnesium, N: nitrogen, P: phosphorous, Zn: zinc.

**Table 3:** Acid binding capacity (ABC) and buffering capacity (BC) of common feed ingredients used in broiler diets, milliequivalent of hydrochloric acid per kg ^1,2,3^.

Ingredient	No. of Samples	ABC ^4^	BC ^5^
Inorganic			
Limestone	7	13,320 (10,723–15,386)	2074 (1589–2639)
Oyster grit	1	12,517	1801
Dicalciumphosphate	1	4661	988
Monocalcium phosphate	1	686	643
Animal-based			
Meat and bone meal	2	1478 (1092–1863)	415 (351–479)
Fish meal	1	1411	554
Meat meal	1	1152	357
Blood meal	1	445	113
Plant-based			
Soybean meal	3	820 (795–839)	210 (197–210)
Canola meal	1	739	204
Sunflower meal	2	727 (718–736)	216 (203–229)
Wheat bran	2	416 (393–439)	104 (90–117)
Peas	1	343	85
Sorghum	2	209 (190–227)	51 (44–57)
Barley	3	186 (176–200)	60 (55–62)
Wheat	5	180 (156–203)	46 (39–53)
Triticale	1	190	46
Maize	1	156	46

^1^ Unpublished data, Massey University. ^2^ Samples were analysed in triplicate. Values in parentheses refer to range of values. ^3^ Measurements were taken at pH 3. ^4^ ABC was calculated by the following formula: ABC (milliequivalent of HCl/kg) = [(Molar concentration of HCl (mole/mL) × volume of acid (mL))/weight of sample (g)] × 1000; ^5^ BC was calculated by the following formula: BC (milliequivalent of HCl/kg) = [ABC/(initial pH − final pH)].

**Table 4:** Calcium content in common feed ingredients used in poultry diets (as fed basis) ^1^.

Ingredient	Calcium (g/kg)
Plant-based	
Cereals:	
Maize	0.2
Wheat	0.5
Rice	0.8
Sorghum	0.4
Barley	0.3
Cereal by-products:	
Wheat bran	1.4
Corn gluten feed (with bran)	4.0
Rice bran	7.0
Vegetable protein sources	
Soybean meal	2.7–2.9
Canola meal	6.8
Sunflower meal	2.1
Animal-based	
Meat and bone meal	103
Fish meal	22.9–51.1
Poultry by-product meal	30
Blood meal	5.0
Feather meal	3.3
Inorganic	
Limestone	380
Oyster shell	380
Dicalcium phosphate	220
Monocalcium phosphate	160

^1^ Adapted from NRC (1994) [74].

**Table 5:** Apparent (AIDC) and true (TIDC) ileal digestibility coefficients of calcium (Ca) for Ca sources in broiler chickens.

Ca Source	Dietary Ca (g/kg)	Ca: npP Ratio	Age (Day)	AIDC	TIDC	Methodology	Reference
Limestone	6.8	1.5	24	0.63	0.65	Direct	[42]
Limestone	9.0	2.0	24	0.56	0.57	Direct	[42]
Limestone	11.3	2.5	24	0.48	0.49	Direct	[42]
Limestone	9.0	2.0	24	0.49	0.50	Direct	[44]
Limestone	9.0	1.1	24	0.36–0.65	0.47–0.55	Direct	[46]
Limestone	9.0	2.0	7–42	0.27–0.53	-	Direct	[47]
Limestone	8.0	2.0	21	0.43, 0.50	-	Direct	[48]
Limestone	9.0	2.0	24	0.57–0.62	-	Direct	[79]
Limestone	3.3	0.9	27	0.56	0.64	Regression	[131]
Limestone	4.3	1.0	27	0.60	0.64	Regression	[131]
Limestone	5.3	1.0	27	0.62	0.64	Regression	[131]
Limestone	6.9	-	11	0.50	-	Direct	[133]
Limestone	6.9	-	25	0.32	-	Direct	[133]
Oyster shell	9.0	2.0	24	0.43	0.44	Direct	[44]
Dicalcium phosphate (DCP)	9.0	1.1	24	0.27, 0.33	0.28, 0.34	Direct	[45]
DCP	2.9–8.6	1.1–1.2	24	-	0.13	Regression	[45]
DCP	8.6	1.2, 2.0	24	-	0.21, 0.25	Difference	[45]
DCP	9.0	1.1	24	0.30	0.32	Direct	[46]
DCP	3.3–5.3	0.9–1.0	27	0.61–0.63	0.67	Regression	[131]
Monocalcium phosphate (MCP)	9.0	0.7	24	0.32	0.33	Direct	[45]
MCP	9.0	0.6–0.7	24	0.41	0.43	Direct	[46]
MCP	7.3	0.7	11	0.77	-	Direct	[133]
MCP	7.3	0.7	25	0.56	-	Direct	[133]
Meat and bone meal (MBM)	8.3	1.9	31	0.40–0.55	0.41–0.56	Direct	[43]
MBM	9.0	1.1	24	0.39	0.41	Direct	[46]
MBM	8.0	1.9	31	0.38–0.56	0.46–0.60	Regression	[109]
Fish meal	9.0	1.4	24	0.23	0.24	Direct	[45]
Poultry by-product meal	9.0	1.5	24	0.28	0.29	Direct	[45]
Canola meal	6.0	2.0	24	0.29	0.31	Direct	[45]
Canola meal	2.2	1.5	21, 42	-	0.64, 0.44	Direct	[134]
Soybean meal	1.4	1.0	21, 42	-	0.54, 0.39	Direct	[134]

npP: nonphytate phosphorous.

**Table 6:** Factors affecting Ca digestibility in poultry.

Factor	Reference
Dietary factors:	
Basal diet composition	[46]
Dietary Ca concentration	[54,59,123,136]
Dietary P concentration	[58,79,137]
Ca:P ratio	[42,54,138,139]
Ca source/origin	[43,79,100,109,111]
Particle size of Ca source	[42,44,89,123,140]
In vitro solubility of Ca source	[79]
Dietary phytate	[23,141]
Phytase	[23,88,134,141,142,143]
Other enzymes or combination of enzymes	[144,145]
Steam conditioning	[146]
Antinutritive factors	[147]
Bird factors:	
Age	[47,133,148,149,150]
Class of chickens	[37,48,151,152]
Physiological factors:	
Ca status	[123]
Dietary adaptation length	[45,46,133]

**Table 7:** The ileal Ca digestibility of Ca sources as affected by particle size, Ca source and in vitro solubility, measured at day 21 or 24 post-hatch.

Ca source	Geometric Mean Diameter (mm) ^1^	In Vitro Solubility ^2^	Apparent ^4^/True ^5^ Ileal Ca Digestibility Coefficient	Reference
Limestone	<0.5 vs. 1–2	0.60 vs. 0.33	0.43 vs. 0.71 ^5^	[42]
Limestone	<0.5 vs. 1–2	0.60 vs. 0.33	0.38 vs. 0.62 ^5^	[44]
Limestone	0.20	0.53–0.60 ^3^	0.54–0.61 ^4^	[79]
Limestone	0.37	0.47	0.51 ^5^, 0.36 ^4^	[46,47]
Limestone	0.46	0.56	0.50 ^4^	[48]
Limestone	1.30	0.23	0.43 ^4^	[48]
Oyster shell	<0.5 vs. 1–2	0.60 vs. 0.37	0.33 vs. 0.56 ^5^	[44]
MBM	0.62–0.88 ^3^	-	0.41–0.56 ^5^	[43]

^1^ Particle size of limestone samples was determined using a set of sieves as described by Baker and Herman [153]. The samples were passed through the sieve stack on shakers for 10 min and the weight of sample retained on each sieve was determined. Particle size was reported as Geometric Mean diameter with Geometric Standard Deviation. ^2^ Zhang and Coon [154] method (200 mL, 0.2 N hydrochloric acid). ^3^ Range is related to number of different sources (three limestone and four MBM [meat and bone meal] samples).

**Table 8:** Reported apparent ileal digestibility coefficients (AIDC) of calcium (Ca) in diets/ingredients at different ages of broilers.

Reference	Ingredients	AIDC					
		Day 20	Day 34	-	-	-	-
[149]	Maize, SBM, limestone, DCP	0.51–0.63	0.35–0.59				
		Day 11	Day 25	-	-	-	-
	Limestone	0.50	0.32				
[133] ^1^	MCP	0.77	0.56				
	SBM	0.66	0.54				
		Day 4	Day 6	Day 8	Day 10	Day 12	Day 8
[155]	Maize, SBM, limestone, DCP	0.56	0.63	0.61	0.62	0.67	0.72
		Day 9	Day 21	-	-	-	-
[150]	Maize, SBM, limestone, MCP	0.53–0.59	0.48–0.59				
		Day 10	Day 27	Day 41	-	-	-
[24]	Maize, SBM, canola meal, wheat bran, DCP, limestone	0.29	0.41	0.31			
		Day 14	Day 35	-	-	-	-
[140]	Maize, SBM, PBPM, limestone	0.56–0.65	0.55–0.48				
		Day 7	Day 14	Day 21	Day 28	Day 35	Day 42
[47] ^2^	limestone	0.51	0.53	0.36	0.34	0.41	0.27

Abbreviation: SBM: soybean meal, DCP: dicalcium phosphate, MCP: monocalcium phosphate, PBPM: poultry byproduct meal. ^1^ Maize-starch-based semipurified diet was used and therefore, the values of AIDC relate to the ingredients. ^2^ Diets were Maize-based and the Ca concentration in maize is negligible. Therefore, the values of AIDC relate to the ingredient (limestone).

**Table 9:** Summary of the standardised ileal digestible (SID) calcium (Ca) and phosphorous (P) requirements for maximum weight gain and tibia ash in Ross 308 broiler starter, grower and finishers.

	Starter (1–10 d) [28]	Grower (11–24 d) [29]	Finisher (25–35 d) [55]
Weight gain			
SID Ca (g/kg)	3.32	3.05	3.50
SID P (g/kg)	5.00	3.50	3.50
SID Ca:SID P	0.66	0.87	1.00
Tibia ash			
SID Ca	4.51	3.69	3.00–3.50
SID P	5.00	3.50	3.50
SID Ca:SID P	0.87	1.05	0.86–1.00
Ross (2019) recommendations ^1^			
SID Ca	4.40 (9.6)	4.03 (8.7)	4.25 (7.8)
SID P	5.40	4.83	3.91
SID Ca:SID P	0.81	0.83	1.09

^1^ Calculated equivalent SID values based on recommendations for total Ca (given in parenthesis, g/kg) and available P.

**Table 10:** Summary of the standardised ileal digestible (SID) calcium (Ca) and phosphorous (P) requirements for maximum tibia ash in Arbor Acres broiler starter, grower and finishers.

	Starter (1–10 d) [50]	Grower (11–24 d) [177]	Finisher (25–42 d) [178]
Weight gain			
SID Ca (g/kg)	3.32	3.05	3.50
SID P (g/kg)	5.00	3.50	3.50
SID Ca:SID P	0.66	0.87	1.00

## Data Availability

All available data incorporated in the manuscript.
